# Medico Legal Insanity

**Published:** 1857-03

**Authors:** 


*Medico-Legal Insanity.*—We had hardly expected to allude to this subject again, but we notice in some of our exchanges a tone of remark which we are sorry to attribute to a feeling of personal hostility to Profs. Parker and Gilman, the medical witnesses in the Huntington trial. We have already expressed our personal opinion as to the unsoundness of that evidence, but we do not see in that any reason why these gentlemen should be followed up by pertinacious and unkindly criticism. Both are above suspicion of corruption, and both, doubtless, testified to what they sincerely believed. And one of them has since published a report of the medical evidence on that trial, which explains to us, satisfactorily, the motives—motives of overflowing kindness and humanity—which governed him. A cooler brain and a sterner heart would reason differently, but we honor the spirit and the intentions of these witnesses, while we differ from their conclusions.

What we regard as the principal error in Prof. Gilman's argument, is his assumption that insanity is a fixed quantity, and that no degree of responsibility is connected with its milder forms. It is as necessary that society should be protected from the passions of the insane as from those of wicked men. And it is known that the fear of punishment is operative with the insane.

We notice that Prof. Dunglison has been quoted as saying that "*medical men are no better judges of the existence of mental alienation than well-informed and discriminating individuals not of the profession.*" The claim of medical testimony to consideration is, that it is "well-informed" on the subject of mental alienation. Now if secular testimony is as good as professional, where is the necessity of "individuals not of the profession," being "well-informed and discriminating?" It strikes us that this *reductio* ad absurdum takes the pith out of Prof. Dunglison's argument, somewhat. If "well-informed" medical testimony is no better than any other, why should we ask that non-professional witnesses should be well informed. It will do them no good!

This subject is environed with difficulties. We should wait for a little before we condemn harshly the motives of good men. The whole question requires a calm, philosophical investigation, and the natural feeling of resentment, engendered in many breasts by the undeserved abuse heaped on these witnesses, will reäct to the creation of stubborn prejudices. This heated, personal argument, is as unsafe as it is unsound, and we, for one, will have nothing to do with it.

We do not recollect, in the course of our experience in medical journalsm, to have seen a greater mass of imperfect and erroneous argument than in this discussion, and what especially annoys us is, that the balderdash is mostly on the right side, which only needs genuine argument. Our cause may pray to be delivered from its friends.

